# Immunization strategies against respiratory syncytial virus in Panama: considerations for decision-making in a tropical country—a working group meeting report

**DOI:** 10.3389/fpubh.2025.1547875

**Published:** 2025-04-02

**Authors:** Xavier Sáez-Llorens, Catherine Lissette Castillo, Danilo Franco, Raúl Esquivel, Kathia Luciani, Rodrigo DeAntonio

**Affiliations:** ^1^Hospital del Niño, Panama City, Panama; ^2^Ministry of Health (Panama), Panama City, Panama; ^3^Gorgas Memorial Institute of Health Studies, Panama City, Panama; ^4^Omar Torrijos Herrera Pediatric Specialty Hospital (CSS), Panama City, Panama; ^5^CEVAXIN Centro de Vacunación e Investigación, Panama City, Panama

**Keywords:** respiratory syncytial virus, passive immunization, maternal vaccine, strategies, Panama

## Abstract

Following the approval of the respiratory syncytial virus (RSV) vaccine and a new monoclonal antibody for passive immunization in children, some Latin American countries have begun using both strategies to prevent RSV-disease. We reviewed these strategies to generate recommendations for their implementation in the Republic of Panama. Experts from different areas and health institutions in Panama gathered in an academic working group to discuss epidemiological data, safety and efficacy aspects, and health economic evaluation elements for both strategies. Aspects of implementing new strategies in Panama were addressed, considering the implementation risks, target population, acceptability, resources, equity, programmatic feasibility, and impact evaluation. According to experts, passive immunization must be universal, including children not exposed to their first RSV season. It could be flexible according to seasonality and must be performed at birth while other vaccines are administered. The immunization must be performed outside the Expanded Program on Immunization (EPI). Maternal vaccination targets all children whose mothers are eligible for vaccination and could receive it within the recommended gestational age (32 weeks) as per the World Health Organization and Pan American Health Organization recommendations. A specific RSV surveillance must be implemented in Panama to measure the impact of the strategy implemented. Likewise, an economic evaluation should be performed to define the cost-effectiveness of these strategies. An education campaign and dissemination of the intervention of the population through healthcare professionals is recommended.

## Introduction

Respiratory syncytial virus (RSV) is a viral agent identified in 1957. RSV generates a significant global burden of disease in terms of cases, hospitalizations, and deaths in high, middle, and low-income countries (1). Annually, there are around 33 million severe cases worldwide, 3 million 600 thousand hospitalizations (it is a leading cause of pediatric hospitalizations), and around 100,000 deaths in children under 6 months of age (1).

By the age of 2 years, most children will have been infected with RSV at least once. Ninety percent of these infections cause mild upper respiratory tract symptoms, but 10% develop lower respiratory tract infections such as bronchiolitis or pneumonia (10–20% will require hospitalization) (2).

Fifty percent of those hospitalized are under 3 months old, and 70% are under 6 months. 1% of hospitalized children die (1). It is estimated that 40–50% of children who die do not access hospital care.

While children with pre-existing conditions are more susceptible to RSV complications, children with no risk factors also die annually from this infectious agent (3). In some countries, the virus occurs seasonally (4), but in countries such as Panama, transmission is continuous, with peaks usually in rainy months (July to November) (5).

The incidence rates of pneumonia, bronchopneumonia, and bronchiolitis for the period 2018 to 2023 in Panama show that children under 1 year and 1 to 4 years old are the most affected, as well as people >65 years old. Pneumonia mortality rates also confirm that these age groups are the most affected. In Panama, the Ministry of Health conducts passive surveillance of respiratory infections focused on influenza and severe acute respiratory infections such as pneumonia, bronchopneumonia and bronchiolitis. So far, there is no specific surveillance for RSV (6), but the notification of a death due to the virus is mandatory.

There are differences between urban areas and areas of difficult access where human resources and access to technology are limited, which leads to a loss of opportunity for notifications. Thus, there may be underreporting of bronchiolitis or bronchopneumonia cases.

The objective of the academic working group was to review the new strategies to prevent RSV infection considering several factors, to generate recommendations for possible implementation in Panama.

### Review of epidemiologic data

The Hospital del Niño, Dr. José Renán Esquivel, as the pediatric reference institution in Panama, conducts epidemiological surveillance of RSV based on the case definition established in the surveillance standard for severe acute respiratory infections (SARI) in Panama (7). Before the pandemic, surveillance showed a seasonal RSV pattern peaking between August and September, with a variable reduction at the end of the year but present throughout the year. Among the risk factors, comorbidities played an important role in the cases admitted to the hospital’s intensive care unit (8).

After the pandemic and during 2021, the seasonal pattern of RSV shifted (9), raising the number of RSV-related cases, although this even occurred in months when RSV was not the most prevalent. However, in 2022 and 2023, it returned to its trend. Information began to be recorded through the molecular biology laboratory and RSV-specific antigenic and polymerase chain reaction (PCR) tests (10).

Regarding the distribution by sex of confirmed RSV cases in the hospital, boys represent 54.7% versus 45.3% of girls. As for the clinical pattern of the confirmed cases on admission to the hospital, 50.7% were due to pneumonia, 37.7% due to bronchiolitis, 9.8% due to URTIs, and 1.4% due to asthma attacks (10).

Coinfections with RSV are present but are not a cause of severity. Healthcare-associated infection (HAIs) rates, combined with RSV rates per minor admitted to the hospital, show that minors are at risk of nosocomial infections, which increases mortality risk, hospital stay, and disease-associated costs. The RSV case fatality rate for hospital admissions for the year 2023 was 1.24% (10).

### Safety and efficacy aspects of the new strategies

Results presented during the working group of a Phase 3 study conducted in 18 countries in the northern and southern hemispheres with nearly 7,000 women in two RSV seasons showed that the maternal vaccine has a satisfactory safety profile. The most frequent adverse events were pain at the injection site and post-vaccination headache and nausea, all of which were mild to moderate and resolved in a short period. Adverse events of special interest (AESIs) included a slight increase in preterm deliveries in women who received the vaccine versus placebo, and an increase in hypertensive disorders of pregnancy; however, the difference was not statistically significant in both cases (11).

The vaccine showed effectiveness in reducing severe lower respiratory tract diseases. Vaccine efficacy for severe medically attended disease at 90 days was 81.8% with a lower interval of 40. At 120 days, efficacy was 73.9%; at 150 days, 70.9%; and at 180 days, 69.4%. For non-severe medically attended disease, vaccine efficacy at 90 days was 57.1% with a lower interval of 14; therefore, this objective was not met (11).

In summary, the vaccine was effective against severe RSV lower respiratory tract cases at 90 days, when the infant is most at risk of severe infection and hospitalization. Protection is prolonged until an infant is 6 months old.

95% of the mothers who participated in the study breastfed their babies for the first 6 months, but there was no difference in vaccine protection between the breastfed and non-breastfed groups (11). This vaccine is also recommended in older adults (12). The maternal vaccine is administered in Latin America in Argentina, Chile, and Brazil. This vaccine specifically targets pregnant women, a population often excluded from vaccine trials (13).

As for the monoclonal antibody, Panama took part in the Melody study conducted with around 3,000 preterm and term infants. Efficacy was between 75 and 85% for severe infection and hospitalization due to RSV. The use of antibiotics was also evaluated, demonstrating a reduction of between 25 and 35% in their use (14). Kaplan Meier curves showed that protection was preserved for 6 months. Evaluation by subpopulations showed that efficacy was similar regardless of place of residence (southern or northern hemisphere), age, sex, race, and weight. In hospitalized children, the disease was shown to be less severe (15).

A study conducted in South Africa showed that the long-acting monoclonal antibody nirsevimab’s protection extended up to almost 1 year, with hospitalization rates of 1.9% versus 3.9% in the placebo group (16). Although children receiving nirsevimab can become infected, they usually do not develop severe diseases. Natural infections can increase antibody production, so children are protected for a longer time. This was demonstrated in a study quantifying post-F antibodies (natural infection against the virus) (17).

The first country to introduce nirsevimab was Spain (15). In Galicia, 97.5% of children under 6 months of age receive the antibody and are monitored weekly with updated data. Hospitalizations for RSV-associated pneumonia or bronchiolitis declined by 80% in the group of infants who received nirsevimab (18). Recent data from Luxembourg and Austria show equal effectiveness (19).

Chile is the first country in the Latin American region to include the monoclonal antibody for all newborns and infants under 6 months from April to May 2024 (20). Country data up to epidemiological week 25 of 2024 with population eligible for nirsevimab show a significant decrease in hospitalizations, hospitalizations in critical patient units and average hospital stays in immunized children compared to previous years (21).

### Economic evaluation aspects to consider

In Panama, the monoclonal antibody palivizumab is approved, and it consists of 5 to 6 doses compared to one dose of nirsevimab (22). In terms of cost, the palivizumab dose ranges from USD 1,200–1,500 per vial (23). The cost of nirsevimab is assumed to be between USD 125 and 300, based on information from cost-effectiveness studies in countries that have introduced nirsevimab (24).

The half-life of palivizumab is approximately 3 weeks compared to about 60–70 days for nirsevimab, which would allow protection beyond 5 months after administration of a single dose (17). An economic evaluation of the new strategies is needed to define whether they would be cost-effective interventions if they were to be universally implemented. Ideally, the use of monoclonal should be compared, determining the time of introduction during the year and whether it should be administered continuously or only in the season with the highest number of cases. Similarly, the economic implications and public health impact of the maternal vaccination strategy must be evaluated, considering the duration of protection, vaccination coverage, and the fact that at least 2 weeks are required to produce the antibodies that will be transferred to the newborn.

The evaluation must also consider total costs and annual resource utilization, outcomes in the burden of disease (total cases, hospitalizations, and deaths) and quality-adjusted life years, and the number of health events prevented, including outpatient consultations, hospitalizations, deaths, and sequelae (recurrent wheezing episodes) from the perspective of the payer, i.e., the health care system (25).

The group of experts agrees that it must be a state policy to favor and maintain the funds allocated to the strategy in the long term.

## Discussion

### Risks of implementing new immunization strategies

RSV is a clear public health issue in Panama (10). Implementing a new intervention for newborns may raise questions among parents, so health personnel must be trained in all the benefits of implementation to clearly explain the advantages to parents.

On the other hand, the maternal vaccination program could present challenges to achieving the desired coverage goals (26, 27). The main barriers are compliance with prenatal care, the reluctance of some sectors within the health sector to adopt these preventive measures, and the lower level of access to prenatal care and growth and development checkups for the indigenous population (5).

Any public health intervention must consider the population that may be most affected, such as children who infect their grandparents or siblings, and the impact this has on the outpatient setting, generating indirect costs for parents/carers due to loss of work and out-of-pocket expenses in a country with a high rate of informal labor, such as Panama.

### The target population for the new strategies

The ideal population to consider in Panama is the entire birth cohort to achieve the most significant benefit in the short and long term, as well as children who have not been exposed to their first season.

If a universal maternal vaccination program is considered, it should include all children under 6 months of age and those older than 6 months who have risk factors. Respiratory viruses, including RSV, could circulate all over the year in Panama, as occurs in other tropical countries. Nonetheless, most of the cases reported are related to the rainy season, which is associated with higher hospital rates and ICU bed occupancy, mainly between July and November. Passive immunization in Panama can be flexible according to seasonality, as it could be administered at birth or in the following months, protecting premature infants and administering an additional dose in the second year to children at risk.

It is also essential to consider the difference between groups of premature infants, children with at-risk conditions, and healthy children (28).

### Acceptability of the immunization strategies

An educational campaign and outreach of the intervention from health professionals to the population is necessary. The acceptability of passive immunization is considered potentially high due to the experience with the administration of palivizumab and with the rest of the vaccines. In the experience of Galicia, Spain, which implemented passive immunization in newborns with the other vaccines administered at birth, high coverage was rapidly achieved, significantly impacting the reduction of hospitalizations due to RSV in its first season (29).

A qualitative study of views and attitudes of pregnant women toward maternal immunization in Panama showed that despite the barriers, there is a high acceptability of maternal immunization among the women interviewed (30). However, some caution may arise regarding immunization strategies during pregnancy, a phenomenon that is observed in a variety of clinical settings and is not limited to obstetricians alone. In addition, prenatal care faces post-pandemic continuity challenges, further complicating the implementation of new vaccination interventions in this group.

### Resources

The investment of using a passive immunization strategy for the entire birth cohort will depend on the price of the new monoclonal and the new maternal vaccine. Currently, the country invests around USD 1,700.000 for palivizumab to cover premature infants with at-risk conditions during the peak RSV circulation season, which accounts for less than 2%. Each child on average uses approximately 2 and a half vials for the 5 recommended doses. The cost of B/1,200 per vial amounts to approximately B/3,000 per child.

Meanwhile, the cost of maternal vaccination per dose is around USD 280 (31).

While the costs of these strategies vary considerably, it is important to consider the impact that they would have on reducing hospitalization costs, sequelae, medication costs, and the effects of the virus on the child in the medium and long term, as they could be used in the largest proportion of children, as has been the experience of universal programs such as rotavirus and *Haemophilus influenzae type B* meningitis.

### Equity

Passive immunization would be best performed at birth, where a high percentage (>90%) of children receive hepatitis B and BCG vaccines. However, when evaluated by health region and, including the three Indigenous communities and the Darien region, professional childbirth care is below 60%. A different type of strategy would be needed in these areas, such as the newborn’s first visit at 8 days of age, to achieve the expected impact.

The issues of education and sustainability of the intervention should be evaluated when introducing the strategy, since one of the problems with indigenous populations is that they are nomadic and therefore, it is difficult to ensure continuity in the interventions administered.

### Program feasibility and impact assessment

Even though it is not a vaccine, nirsevimab is stored and administered as such. The logistics of the administration of this monoclonal should use the experience with palivizumab as a reference so that the medicine area can plan the process for administering passive immunization universally.

It is necessary to establish the passive immunization scenario with nirsevimab outside the Expanded Program on Immunization (EPI), possibly through hospitals directly, to obtain records, high coverage and measurable data, using the logistics currently used in the palivizumab program.

In the case of maternal vaccination, the availability of financial resources, storage, cold chain and distribution with the PAI and its sustainability over time should be confirmed. It should also be confirmed whether the process would be carried out through the PAHO Revolving Fund and/or directly through Farmacias y Drogas, the Panamanian regulatory agency. Maternal vaccination should employ the information system currently used for other vaccines administered during pregnancy, and ideally ensure timely recording so that coverage can be monitored. Co-administration with other vaccines during pregnancy is also recommended (13).

If implemented, it will be necessary to review the sustained supply with the manufacturers of these immunization strategies to ensure the continuity of the program to achieve the highest expected impact.

It is necessary to structure surveillance to detect and measure the impact of any implemented strategy, conduct outpatient surveillance, and strengthen the capacity of sentinel laboratories to detect RSV and evaluate possible genomic changes that may arise in circulating strains once the strategy is implemented.

Neither of the two interventions has completed the registration process with the Panamanian regulators yet, but it is expected to begin soon. The analysis related to both immunization strategies has been presented preliminarily at two conferences (32, 33).

### SWOT analysis

**Figure 1:**
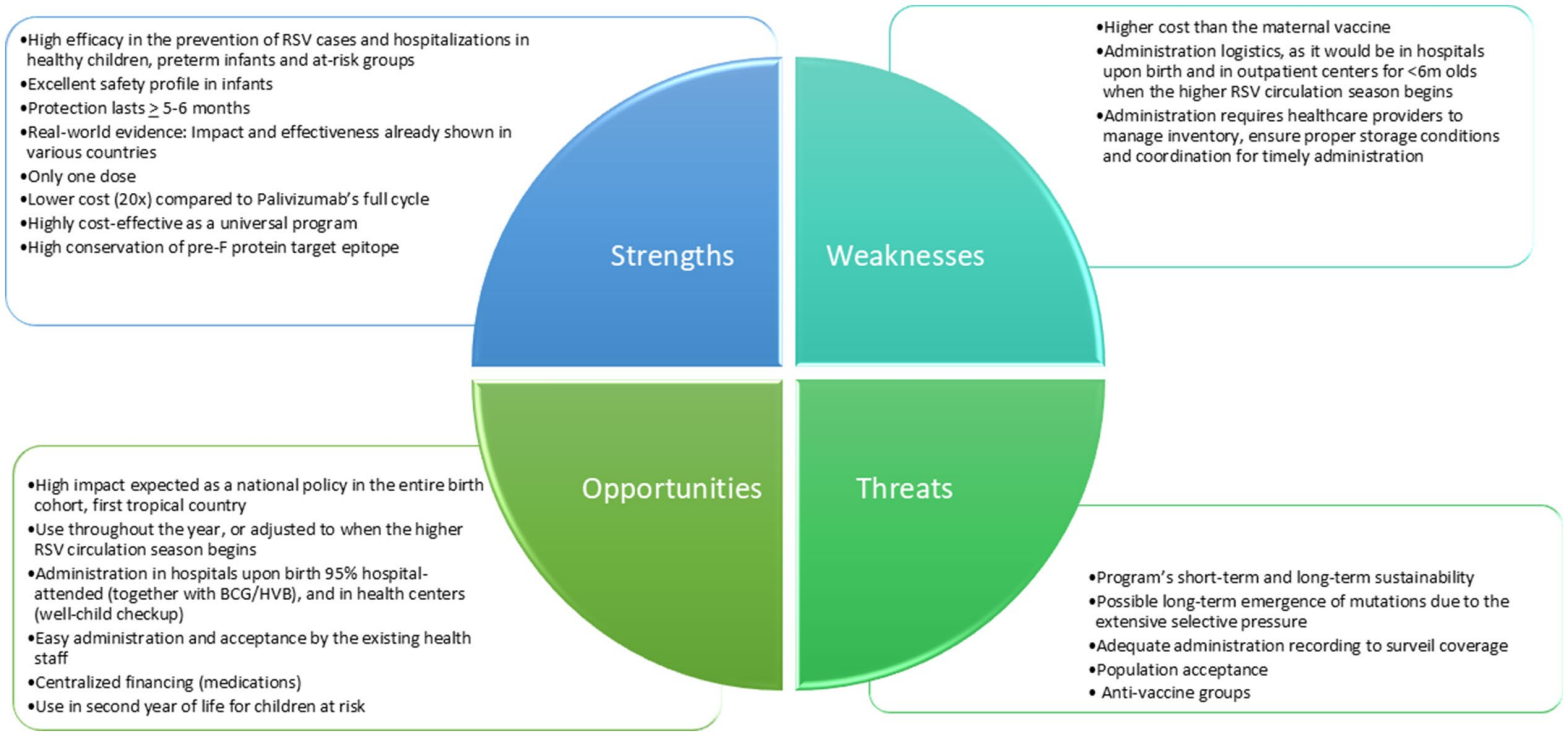
SWOT analysis of nirsevimab monoclonal antibody in the context of the population of the Republic of Panama.

**Figure 2:**
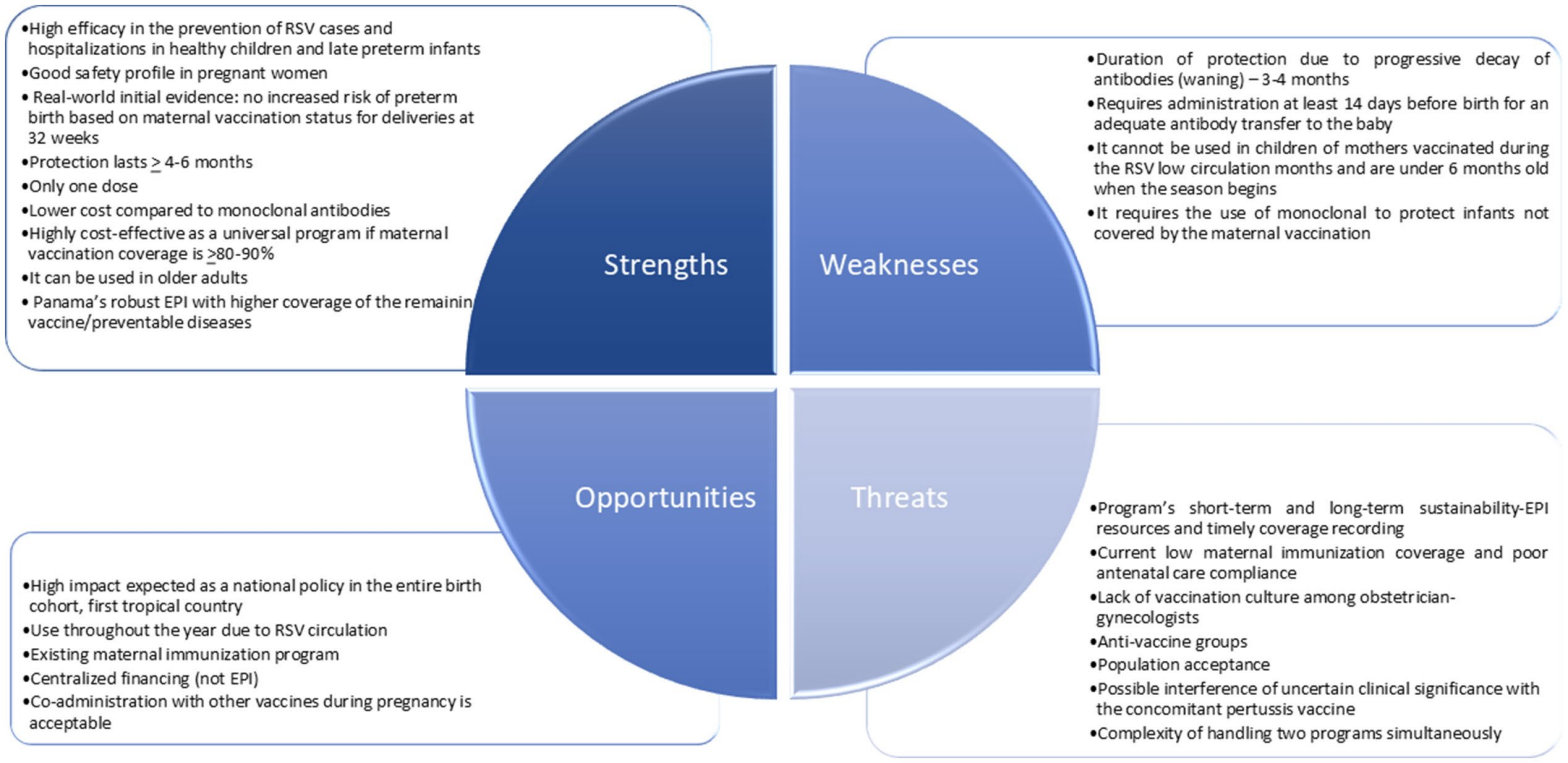
SWOT analysis of the RSV maternal vaccine in the context of the population of the Republic of Panama.

Figure 1 (SWOT analysis of Nirsevimab monoclonal antibody in the context of the population of the Republic of Panama) and Figure 2 (SWOT analysis of the RSV maternal vaccine in the context of the population of the Republic of Panama) provide an analysis of the strengths, weaknesses, opportunities, and threats of both strategies.

## Conclusion

After 70 years of identifying RSV as the leading annual cause of morbidity and mortality in infants, science was indebted to the child population for not having a safe and effective preventive strategy to reduce the burden of disease caused by this virus. The academic working group carried out by the expert group on the aspects to be considered for the introduction of a new RSV immunization strategy in Panama is a starting point that the country’s regulatory authorities may use for its prompt implementation. The potential impact of implementing the recommendations issued in Panama may provide relevant data for other countries or tropical regions where RSV circulation occurs during every month of the year.

## Data Availability

The original contributions presented in the study are included in the article/supplementary material, further inquiries can be directed to the corresponding author/s.
